# A Tribute to Gopi Menon

**DOI:** 10.1002/pne2.12016

**Published:** 2020-04-11

**Authors:** Elaine M. Boyle

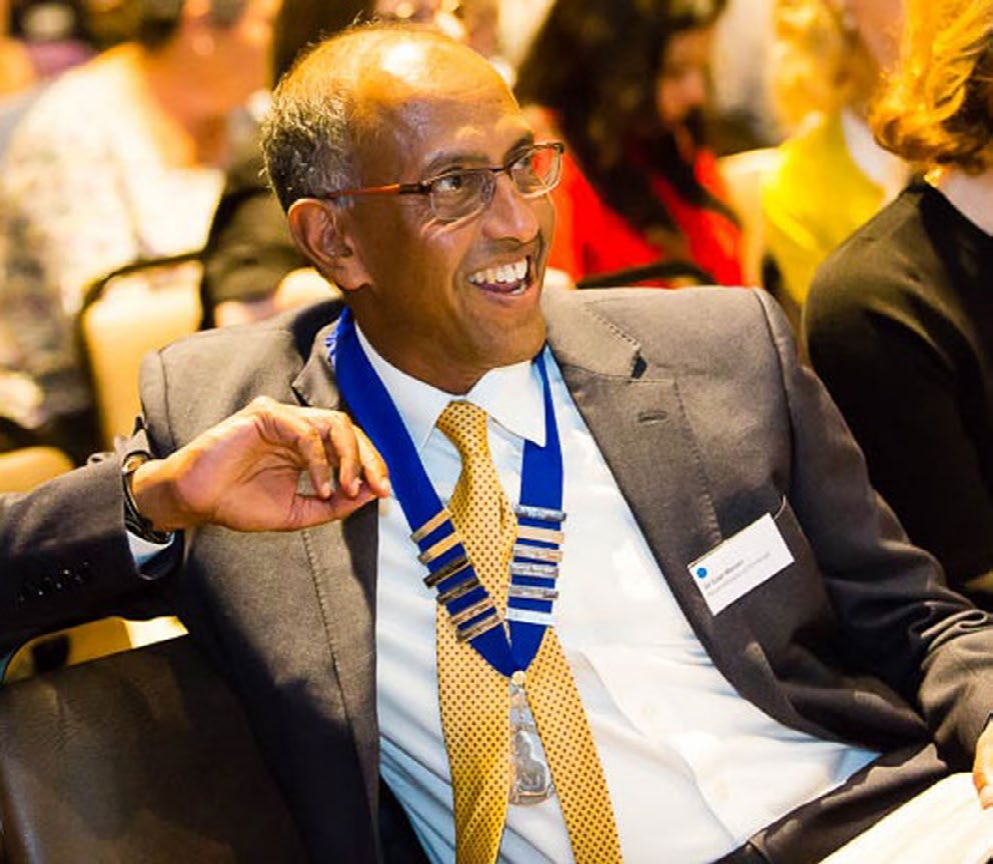

**Gopi Menon MD, FRCP, FRCPCH, FBAPM (Hon)**

**Consultant Neonatologist, Royal Infirmary of Edinburgh**

**Editorial Board Member, Paediatric and Neonatal Pain**

**Born 22 January 1955, Kerala, South India**

**Died 21 August 2019, Edinburgh, UK**

Gopikumar Menon moved from India to Yorkshire, England, with his family, at the age of 9 years and completed his schooling before undertaking preclinical medical undergraduate training at Fitzwilliam College, Cambridge. He completed clinical training at St Mary’s Hospital Medical School and graduated in 1979.

Gopi trained as a pediatrician, working in St Mary’s Hospital in London, Alder Hey Children’s Hospital in Liverpool, Guy’s and Great Ormond Street Hospitals in London. His chosen subspecialty was neonatology, and his neonatal training was completed in the East Midlands of England.

In 1988, Gopi moved to work in Edinburgh as a senior registrar, and in 1994, was appointed as a consultant neonatologist at the Simpson Memorial Maternity Hospital in Edinburgh. He led and delivered high‐quality care for the very large numbers of babies that passed through both the old and new neonatal units in Edinburgh during his 25 years there, and for this, he is appreciated and will be remembered by scores of parents and families. He was an excellent teacher, a fact to which the many students and junior doctors who benefitted from his wisdom, including myself, will testify.

Gopi was a sensitive, thoughtful, committed, and caring clinician, who believed passionately in improving the quality of care delivered to our most vulnerable babies. He had a keen interest in research, as a means of determining evidence to guide best practice in many areas, but particularly in infant nutrition and neonatal pain management.

From a personal perspective, I had the great good fortune to work closely with Gopi during the time I spent in Edinburgh as a postgraduate MD and later PhD student and have many fond memories. He had a keen sense of humor. Discussions with Gopi were always both entertaining and thought provoking. He could be relied upon to question and challenge conventional received wisdom, always seeking to see things from a different and novel angle, and often playing “devil’s advocate”; yet his conclusions were always balanced, fair, and robust. Gopi was part of a number of important projects investigating pain in the newborn, including the NoPain, Neopain, and NeoOpioid collaborations, and he co‐authored multiple papers addressing neonatal pain assessment and management.

Gopi was a supportive colleague, a loyal friend, and a valued advisor throughout the more senior years of my training and when I became a consultant myself. Right until the end of his life, he looked out for me, was a source of encouragement and a great sounding board; we continued to share thoughts and ideas, and he was always interested in, and supportive of my work and achievements.

When I took on the role of Editor‐in‐Chief for Paediatric and Neonatal Pain in 2018‐19, Gopi was an obvious choice to invite to be part of the new Editorial Board. However, he had become President of the British Association of Perinatal Medicine (BAPM), an important and demanding national leadership role. I expected him to be too busy, and indeed, he asked to take a little time to decide whether he would be able to devote the necessary time to meaningfully contribute to the journal, before modestly saying that he was “happy to take on the challenge.”

Sadly, Gopi soon learned that there were far greater challenges ahead when he fell ill in February 2019 and was diagnosed with pancreatic cancer. He continued to take a keen interest in colleagues, in neonatal care and in the work of BAPM throughout his short illness, but unfortunately, the Editorial Board of Paediatric and Neonatal Pain was never able to benefit from the unique input that I am sure he would have brought.

Gopi is very much missed by his many friends, colleagues, and collaborators. He is survived by his wife, Val, and daughters, Natasha and Jessica.

